# Risk Associated with Bee Venom Therapy: A Systematic Review and Meta-Analysis

**DOI:** 10.1371/journal.pone.0126971

**Published:** 2015-05-21

**Authors:** Jeong Hwan Park, Bo Kyung Yim, Jun-Hwan Lee, Sanghun Lee, Tae-Hun Kim

**Affiliations:** 1 Acupuncture, Moxibustion and Meridian Research Group, Korean Institute of Oriental Medicine, Daejeon, South Korea; 2 Division of Cardiovascular and Rare Diseases, Center for Biomedical Science, National Institute of Health, Cheongju, Chungcheongbuk-do, South Korea; 3 Korean Medicine Clinical Trial Center, Korean Medicine Hospital, Kyung Hee University, Seoul, South Korea; Central South University, CHINA

## Abstract

**Objective:**

The safety of bee venom as a therapeutic compound has been extensively studied, resulting in the identification of potential adverse events, which range from trivial skin reactions that usually resolve over several days to life-threating severe immunological responses such as anaphylaxis. In this systematic review, we provide a summary of the types and prevalence of adverse events associated with bee venom therapy.

**Methods:**

We searched the literature using 12 databases from their inception to June 2014, without language restrictions. We included all types of clinical studies in which bee venom was used as a key intervention and adverse events that may have been causally related to bee venom therapy were reported.

**Results:**

A total of 145 studies, including 20 randomized controlled trials, 79 audits and cohort studies, 33 single-case studies, and 13 case series, were evaluated in this review. The median frequency of patients who experienced adverse events related to venom immunotherapy was 28.87% (interquartile range, 14.57–39.74) in the audit studies. Compared with normal saline injection, bee venom acupuncture showed a 261% increased relative risk for the occurrence of adverse events (relative risk, 3.61; 95% confidence interval, 2.10 to 6.20) in the randomized controlled trials, which might be overestimated or underestimated owing to the poor reporting quality of the included studies.

**Conclusions:**

Adverse events related to bee venom therapy are frequent; therefore, practitioners of bee venom therapy should be cautious when applying it in daily clinical practice, and the practitioner’s education and qualifications regarding the use of bee venom therapy should be ensured.

## Introduction

Bee venom is one of the most commonly encountered animal venoms and consists of various chemical agents that induce allergic reactions in the human body [1]. Bee venom therapy (BVT), in which bee venom is used for medicinal purposes, is available worldwide, but is primarily utilized in Asia, Eastern Europe, and South America [2]. The diverse therapeutic applications of BVT include various musculoskeletal conditions, such as arthritis and rheumatism, chronic recalcitrant neuralgia, arthralgia, and immune-related diseases. BVT is also used to desensitize patients to bee stings and thus inhibit allergic reactions [3] [4] [5].

Although the therapeutic utility of bee venom has been demonstrated, its safety profile is an important limiting consideration, because immune responses to BVT can range from trivial skin reactions that resolve over several days to life-threatening responses such as anaphylaxis [6] [7]. In a recent survey, the incidence of systematic reactions (SRs) in patients who received venom and inhaled-allergen subcutaneous immunotherapy was 13.60%, whereas the prevalence of SRs in patients that received bee venom immunotherapy (VIT) was 28.72% [8]. In another survey, 12.13% patients who received VIT experienced SRs (an average of 1.91 SR events per subject), suggesting that serious adverse events (SAEs) due to BVT are quite common [9].

The most significant issue related to the AEs of BVT is that the occurrence of SAEs is unpredictable. It is therefore necessary to determine the prevalence and nature of AEs related to various types of BVT, so that bee venom can be used safely in clinical practice. The aim of this systematic review was to provide summary information regarding the types of AEs related to BVT and their prevalence in treated patients.

## Methods

### Study selection

#### Types of studies

All types of clinical studies, including randomized controlled trials (RCTs) and randomized crossover trials, as well as observational studies, including cohort studies, case-control studies, case series, and case studies, were included in this study.

#### Types of participants

The subjects of the studies evaluated in this review included adults and pediatric patients, and the selection was not limited to studies of patients with specific diseases. Regardless of a patient’s condition and disease status, studies were included if bee venom was used as a key intervention, and AEs that may have been causally related to BVT were reported.

#### Types of interventions

In this review, we included studies of bee sting acupuncture (BSA), a subcutaneous or intramuscular injection of bee venom for the purpose of acupoint stimulation (bee venom acupuncture [BVA], sweet bee venom [SBV]), and dried honeybee venom (apitoxin injections), as well as subcutaneous VIT for desensitization of venom immune reactions. BSA, BVA, SBV, and apitoxin injections usually involve the use of venom derived from bees (family Apidae), whereas VIT generally involves the use of venom from bees (family Apidae) and wasps (family Vespidae) concomitantly. Therefore, we included all types of venom therapy including both bee and wasp venoms. We also reviewed studies where bee venom was used alone or in combination with other treatments. However, studies describing bee stings resulting from random encounters (e.g., during resting or by attack), sting challenge tests, sublingual VIT, and irrelevant venom types were excluded from this study. We included RCTs comparing BVT with no treatment, normal saline injections, and conventional medications for relative risk assessment. Trials in which different types of BVT were compared with each other were excluded.

#### Types of outcome measures

The major aim of this review was to identify the frequency and types of AEs related to BVT. In case studies and case review series, the type of AE was classified into 1 of 3 categories: SR, skin problem (SP), and other (nonspecific reaction, symptom, or sign that was not an SR or SP). If an SR occurred as an AE, it was classified into 1 of 5 categories based on the Mueller classification (grade I, grade II, grade III, and grade IV) [10]. The causal relationship between BVT and AEs was also assessed in each study according to the WHO-UMC causality scale [11]. AEs were scored as certain when they clearly occurred after BVT, disappeared after withdrawal, and could not be explained by other diseases or treatments. AEs were scored as probable when the timing of the AEs and BVT indicated that they were most likely related, they disappeared as a probable result of the discontinuation of BVT, and the events were not induced by other diseases or treatment. AEs were scored as possible when they occurred after BVT treatment but no information was available on the relationship between their disappearance and the withdrawal of BVT and when they could potentially be explained by other diseases or treatments. In addition, AEs were scored as unlikely when the event and the BVT had an improbable causal relationship. AEs were scored as conditional/unclassified when the event occurred but more data were necessary for a conclusion to be reached. Finally, AEs were scored as unassessable/unclassifiable when they could not be evaluated properly owing to insufficient and/or contradictory information [12].

In audits and cohort studies, AE types were divided into SR, large local reaction (LLR), local reaction (LR), and other (nonspecific reaction, symptom, or sign that was not an SR, LLR, or LR). An LLR was defined as swelling exceeding 10 cm in diameter and lasting longer than 24 h, and an LR was defined as local pruritus, edema, or erythema [13]. Finally, the prevalence of AEs related to BVT was assessed through observational studies, including audits and cohort studies.

### Data sources

The following 12 databases were searched: PubMed, EMBASE, the Cochrane Library, CINAHL, China National Knowledge Infrastructure (CNKI), Wanfang (China), Weipu (China), KoreaMED, the Korean Medical Database (KMBASE), the Korean Studies Information Service System (KISS), National Discovery for Science Leaders (NDSL) (Korea), and the Oriental Medicine Advanced Searching Integrated System (OASIS) (Korea). Bibliographic references in relevant publications (Journal of Pharmacopuncture) were manually searched to avoid missing eligible articles. The References sections of reviews on AEs of BVT were searched manually, and articles published through June 2014 were included. The search terms consisted of two parts: “BVT” (e.g., bee sting, apitoxin, or venom immunotherapy) and “adverse events” (e.g., adverse reaction, side effects, risk, or safe). The search strategy was modified appropriately according to the databases. The detailed search strategies for PubMed, China National Knowledge Infrastructure (CNKI), Wanfang (China), Weipu (China), KoreaMED, the Korean Medical Database (KMBASE), the Korean Studies Information Service System (KISS), National Discovery for Science Leaders (NDSL) (Korea), and the Oriental Medicine Advanced Searching Integrated System (OASIS) (Korea) are presented in the Supporting Information.

### Data collection and analysis

#### Study selection

Two independent reviewers (JHP and BKY) screened the articles for inclusion by title and abstract. If disagreements regarding the selection of a study could not be resolved through discussion, the final decision was made by the arbiter (THK).

#### Data extraction and management

One reviewer (JHP) read the full text of the articles selected for review and extracted the data using a standard data extraction form. Another reviewer (BKY) rechecked the data to ensure that it had been extracted appropriately. Any disagreement among the reviewers was resolved by discussion or by the arbiter (THK).

#### Quality assessment of AEs in RCTs

To evaluate the quality of the detection and reporting of the AEs in the included RCTs, 7 items were assessed according to the CONSORT recommendations for harm data: (1) mention of AEs in the title or abstract, (2) mention of BVT-related AEs in the introduction, (3) predefined definition of AEs related to BVT, (4) collection or monitoring method for AEs, (5) mention of the method for analyzing and presenting AEs, (6) mention of any patients who dropped out of the study owing to AEs, and (7) mention of the specific denominator for the analysis of AEs [14] [15]. The quality of each item was judged as good, moderate, bad, or not reported [12]. The quality of a study was scored as good if each item was reported clearly in the manuscript or in the registered protocol. If each item was reported, but not in detail, the methodological quality was scored as moderate. The quality of a study was scored as bad when any of the items were not appropriately reported. If an item was not described at all, it was recorded as not reported.

### Statistical analysis

A meta-analysis of the RCTs was conducted if the incidence of AEs was clearly reported and the relative risk of AEs could be assessed because of similar study designs and intervention methods, including BVT types and control interventions, with minimal clinical heterogeneity. The relative risk of BVT and control interventions was assessed, and effects were calculated using Revman 5.2 software (http://ims.cochrane.org/revman).

## Results

**Fig 1:**
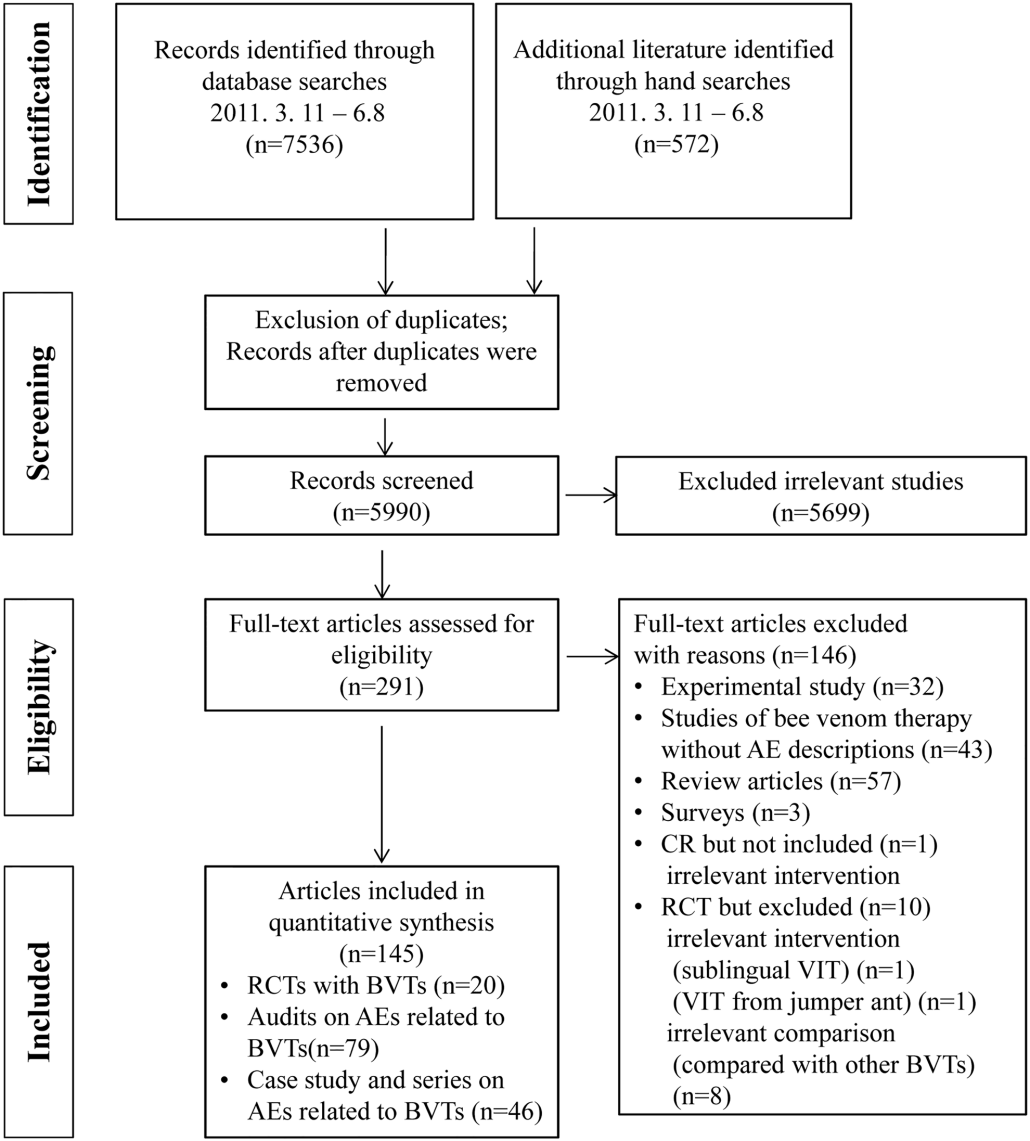
Flow diagram of the study selection process.

Through electronic and manual searching, 8,108 potentially relevant articles were identified, including 5,504 records from PubMed, EMBASE, the Cochrane Library, and CINAHL; 468 records from the Chinese databases; and 2,136 records from the Korean databases, from which 2,118 duplicate records were removed. Through a screening process involving the use of the titles and abstracts of identified records, we excluded 5,699 records that did not meet the inclusion criteria. The remaining 291 articles were reviewed for eligibility, and 146 articles were excluded, including experimental studies (32), reviews (57), surveys (3), studies without description of the assessment of AEs (43), and studies without relevant intervention or comparison groups (11). Finally, 145 studies, including 20 RCTs, 79 audits and cohort studies, 33 single-case studies, and 13 case series, were included in the review (Fig 1).

### Case studies and case series

Thirty-three single-case studies and 13 case series were identified as described in Table 1 [2,7,16–59]. A total of 69 individual isolated cases were reported in 46 papers. Incidents were reported in 11 countries: Korea (37 cases), China (10 cases), the United States (7 cases), France (6 cases), Germany (2 cases), Turkey (2 cases), Canada (1 case), Italy (1 case), Russia (1 case), Saudi Arabia (1 case), and the Slovak Republic (1 case). The reported BVT methods included BSA (29), BVA (21), and VIT (19). Among the 69 AE cases, 58 cases were related to BVT, 6 cases were related to wasp venom treatment, and 5 cases were related to treatment with a mixture of bee venom and wasp venom. Among the 58 AE cases related to treatment with bee venom only, 30 SRs, 23 SPs, and 5 other cases, including cough; headache; uremia; anorexia; discoloration of the sclera; jaundice; painful cyclic uterine contractions; severe pain affecting the left shoulder, chest wall, and left arm; and muscular weakness in the left arm and hand, were reported. The 30 SRs related to treatment with bee venom only were classified as grade I (5 cases), grade II (10 cases), grade III (14 cases), and grade IV (1 case). The severity of the AEs related to BVT only were reported as moderate (34 cases) or severe (24 cases), and the causality was deemed to be probable for 49 cases and possible for 9 cases. Most practitioners were qualified practitioners (30 cases), and 4 patients were treated by unqualified personal with no medical training or licensure regarding BSA. One patient died after treatment by an unqualified BSA practitioner. In 23 cases, there was no description of the practitioner. A pre-treatment skin test for venom allergies was reported in only 10 cases, and it was almost always performed prior to VIT, whereas in most cases of BSA and BVA, it was not reported whether or not this test was conducted.

**Table 1:** Case studies and case series on adverse events associated with bee venom therapy.

Study (first author, year)	Country	Number of cases	Reason for BVT	Practitioner type	BVT stimulation feature	Venom type^a^	Skin test	Injection amount	Concomitant treatment	AE symptoms	AE severity^b^	AE type^c^	Mueller classification^d^	Diagnosis	Causality^e^
**Bee sting acupuncture (BSA) and bee venom acupuncture (BVA)**															
Alqutub 2011 [2]	Saudi Arabia	1 case (F/35)	Multiple sclerosis	Local practitioner	BSA	Bees	Not reported	10 bee stings	Not reported	Fatigue, anorexia, and discoloration of sclera (jaundice)	Severe	Others	-	Hepatotoxicity	Probable
An 2001 [16]	Korea	3 cases a) F/58	a) Degenerative knee arthritis	a) KMD	a) BVA	Bees	a) Not reported	a) BV injection 2,000:1, 0.35 mL	a) Cold pack	a) Extreme pain, muscular convulsion and tremble, ocular hyperemia, sleepiness, stiffness of limbs, and hyperventilation	a) Severe	a) SR	a) Grade III	a) Pain shock	a) Probable
		b) F/57	b) Progressive bulbar paralysis	b) KMD	b) BVA	Bees	b) Not reported	b) BV injection 2,000:1, 0.1 mL	b) Cold pack, acupuncture, pharmacopuncture	b) Extreme pain, facial sweating, asthenia of limbs, pallor face, weak voice, and sleepiness	b) Severe	b) SR	b) Grade III	b) Pain shock	b) Probable
		c) F/54	c) Amyotropic lateral sclerosis	b) KMD	c) BVA	Bees	c) Not reported	c) BV injection 2,000:1, 0.3 mL	c) Cold pack	c) Extreme pain, facial sweating, asthenia of limbs, pallor face, weak voice, and sleepiness	c) Severe	c) SR	c) Grade III	c) Pain shock	c) Probable
Bae 2009 [17]	Korea	1 case (M/76)	Palpable subcutaneous nodule	Not reported	BSA	Bees	Not reported	Not reported	Not reported	Two erythematous plaques, skin ulcerations, and necrosis	Moderate	SP	-	Foreign bodygranuloma	Probable
Cheng 2004 [18]	China	2 cases a) M/2	a) Repeated respiratory infections	a) MD	a) BSA	Bees	a) Not reported	a) 1 bee sting	a) Not reported	a) Arrhythmia,pallor face, nausea, vomiting, and cold sweats	a) Moderate	a) SR	a) Grade II	a) Anaphylaxis	a) Probable
		b) M/3	b) Repeated respiratory infections	b) MD	b) BSA	Bees	b) Not reported	b) 1 bee sting	b) Not reported	b) Arrhythmia,pallor face, nausea, vomiting, and cold sweats	b) Moderate	b) SR	b) Grade II	b) Anaphylaxis	b) Probable
Cho 2010 [19]	Korea	1 case (F/37)	Lower back pain	KMD	BVA	Bees	Not reported	Not reported	Not reported	Skin rash, pruritus, arthralgia,fever, and myalgia	Moderate	SR	Grade I	Serum sickness reaction	Probable
Herr 1999 [20]	Korea	1 case (M/64)	Knee arthralgia	Unqualified person	BSA	Bees	Not reported	Not reported	Not reported	Localized edema and pruritus; skin nodules	Moderate	SP	-	Eosinophilic granuloma	Probable
Huh 2008 [21]	Korea	1 case (M/71)	Knee pain	Not reported	BSA	Bees	Not reported	Not reported	Not reported	Dysarthria, dizziness, and left hemiparesis	Severe	SR	Grade III	Pontine and thalamic infarction	Possible
Jung 2012 [22]	Korea	1 case (F/65)	Knee pain	Unqualified person (apitherapist)	BSA	Bees	Not reported	Not reported	Not reported	Nausea, dizziness, weakness, generalized paresthesia, whole-body wheal, diffuse edema, unconsciousness, and death	Severe	SR	Grade IV	Anaphylaxis, disseminated intravascular coagulation (DIC)	Probable
Karapata 1961 [23]	Russia	1 case (M/51)	Hypertensive disorders	Not reported	BVA	Bees	Not reported	Not reported	Not reported	Vomiting, headache, and uremia	Severe	Others	-	Toxic pulmonary edema	Possible
Kim 2005 [24]	Korea	1 case (F/53)	Pain in the scapular region	KMD	BVA	Bees	Not reported	Not reported	Not reported	Localized pruritus and multiple erythematous papules	Moderate	SP	-	Hypersensitivity	Probable
Kim 2007 [25]	Korea	1 case (F/28)	Not reported	Not reported	BVA	Bees	Not reported	Not reported	Not reported	Facial and generalize edema, backache, and abdominal distension	Moderate	SR	Grade II	Minimal change, nephrotic syndrome	Probable
Kim 2010 [26]	Korea	1 case (F/36)	Knee osteoarthritis	KMD	BSA	Bees	Not reported	Not reported	Not reported	Two erythematous plaques and nodules; skin ulcerations	Moderate	SP	-	Foreign body granuloma	Probable
Kim 2011 [27]	Korea	1 case (F/75)	Knee and lower back pain	KMD	SBV and BVA	Bees	Not reported	SBV injection 2.4 mL, BV injection 4,000:1, 1.0 mL	Pharmacopuncture	Facial erythema localized erythema generalized pruritus chest discomfort mild dyspnea	Moderate	SR	Grade II	Anaphylaxis	Probable
Kwon 2009 [28]	Korea	2 cases a) M/76	a) Lower back pain, knee osteoarthritis	a) KMD	a) SBV	Bees	a) Not reported	a) Not reported	a) Not reported	a) Tongue edema, dysarthria, mild dyspnea, localized erythema, and swelling	a) Severe	a) SR	a) Grade III b) Grade I	a) Anaphylaxis	a) Probable
		b) F/50	b) Pain in hand and shoulder joints	b) KMD	b) SBV	Bees	b) Not reported	b) SBV injection 2.2 mL	b) Pharmacopuncture	b) Generalized pruritus and fever	b) Moderate	b) SR	b) Grade I	b) Anaphylaxis	b) Probable
Lee 1996 [29]	Korea	1 case (F/43)	Chronic eczema-like dermatosis	Not reported	BSA	Bees	Not reported	Not reported	Not reported	Multiple erythematous plaques and nodules	Moderate	SP		Foreign body granuloma	Probable
Lee 1996 [30]	Korea	1 case (F/42)	Polyarthralgia	Not reported	BSA	Bees	Not reported	Not reported	Not reported	Localized edema and redness; subcutaneous nodules	Moderate	SP		Foreign body granuloma	Probable
Lee 2000 [31]	Korea	1 case (M/28)	Ankle sprain	KMD	BVA	Bees	Not reported	Not reported	Not reported	Neck stiffness, chest pressure sensation, stridor, and dyspnea	Severe	SR	Grade III	Anaphylaxis	Probable
Lee 2010 [32]	Korea	1 case (M/59)	Lipoma	Not reported	BSA	Bees	Not reported	Not reported	Not reported	Single erythematous plaques	Moderate	SP	-	Foreign body granuloma	Probable
Lee 2011 [33]	Korea	2 cases a) F/53	a) Knee and lower back pain	a) MD	a) BVA (apitoxin injection)	Bees	a) Not tested	a) Not reported	a) Not reported	a) Multiple erythematous plaques and nodules, skin ulcerations, and tenderness	a) Moderate	a) SP	a) -	a) Foreign body granuloma	a) Probable
		b) M/59	b) Foot pain	b) MD	b) BVA (apitoxin injection)	Bees	b) Not tested	b) Not reported	b) Not reported	b) Multiple erythematous plaques and nodules, skin ulcerations, and tenderness	b) Moderate	b) SP	b) -	b) Foreign body granuloma	b) Probable
Lee 2013 [34]	Korea	1 case (M/50)	Back pain	KMD	BSA	Bees	Not reported	Not reported	Not reported	Multiple erythematous plaques and nodules	Moderate	SP	-	Chronic folliculitis and granuloma	Probable
Li 2002 [35]	China	1 case (F/63)	Limb joint pain	MD	BSA	Bees	Not reported	More than 20 bees stings	Not reported	Pallor face, chest discomfort, dyspnea, dysarthria	Severe	SR	Grade III	Anaphylaxis	Probable
Li 2005 [36]	China	4 cases a) F/67	a) Rheumatoid arthritis	a) Not reported	a) BSA	Bees	a) Not reported	a) 3 bee stings	a) Not reported	a) Generalized pruritus, large amounts of sweat, pallor lip, decreased consciousness, hot feeling of the extremities, chest discomfort, and nausea	a) Severe	a) SR	a) Grade III	a) Anaphylaxis	a) Probable
		b) F/63	b) Rheumatoid arthritis	b) Not reported	b) BSA	Bees	b) Not reported	b) Not reported	b) Not reported	b) Pallor pace (blue violet), tachypnea, dysarthria, and dizziness	b) Severe	b) SR	b) Grade III	b) Anaphylaxis	b) Probable
		c) F/59	c) Rheumatoid arthritis	c) Not reported	c) BSA	Bees	c) Not reported	c) 2 bee stings	c) Not reported	c) Localized edema and redness, and generalized urticaria	c) Moderate	c) SR	c) Grade I	c) Anaphylaxis	c) Probable
		d) F/36	d) Rheumatoid arthritis	d) Not reported	d) BSA	Bees	d) Not reported	d) 2 bee stings	d) Not reported	d) Systemic papules, generalized pruritus, localized edema, and redness	d) Moderate	d) SR	d) Grade I	d) Anaphylaxis	d) Probable
Park 1998 [37]	Korea	1 case (F/52)	Facial papule	Self	BSA	Bees	Not reported	Not reported	Not reported	Ulcerative tumor	Moderate	SP	-	Eosinophilic foreign body granuloma	Probable
Park 2000 [38]	Korea	1 case (M/50)	Not reported	Not reported	BVA	Bees	Not reported	Not reported	Not reported	Severe diaphoresis, dizziness, palpitation, dysarthria, and left hemiparesis	Severe	SR	Grade III	Ischemic stroke	Probable
Park 2013 [7]	Korea	2 cases a) F/44	a) Arthralgia pain	a) Not reported	a) BSA	Bees	a) Not reported	a) Not reported	a) Not reported	a) Ulcerative tumor	a) Moderate	a) SP	a) -	a) Live bee acupuncture dermatitis	a) Probable
		b) M/10	b) Eczema	b) Not reported	b) BSA	Bees	b) Not reported	b) Not reported	b) Not reported	b) Whitish plaques with erythematous papules	b) Moderate	b) SP	b) -	b) Live bee acupuncture dermatitis	b) Probable
Rhee 2009 [39]	Korea	1 case (M/49)	A small nodule	Not reported	BVA	Bees	Not reported	Not reported	Not reported	Erythematous tumor	Moderate	SP	-	Giant dermatofibroma	Probable
Rho 2009 [40]	Korea	1 case (F/49)	Knee arthritis	Not reported	BVA	Bees	Not reported	Not reported	Not reported	Fever, dysuria, face edema, and generalized erythematous; popular rash	Moderate	SR	Grade I	Systemic lupus erythematosus	Possible
Shim 2011 [41]	Korea	1 case (M/52)	Paralysis	KMD	BVA	Bees	Not reported	Not reported	Not reported	Multiple erythematous plaques and nodules, skin ulcerations, and tenderness	Severe	SP	-	*Mycobacterium chelonae* infection	Probable
Song 2002 [42]	Korea	2 cases a) F/42	a) Pain in the scapular region	a) Unqualified person	a) BSA	Bees	a) Not reported	a) Not reported	a) Not reported	a) Generalized urticaria, facial edema, dyspnea, and chest pain	a) Severe	a) SR	a) Grade II	a) Anaphylaxis	a) Probable
		b) F/39	b) Lower back pain	b) Unqualified person	b) BSA	Bees	b) Not reported	b) Not reported	b) Not reported	b) Facial edema, generalized urticaria, pruritus, lower abdomen pain, and dyspnea	b) Severe	b) SR	b) Grade II	b) Anaphylaxis	b) Probable
Veraldi 1995 [43]	Italy	1 case (M/65)	Spinal column arthrosis	Not reported	BSA	Bees	Not reported	Not reported	Not reported	Swelling, edema, and numerous inflammatory nodules	Severe	SP	-	Long-lasting subacute inflammatory reaction	Probable
Yoo 1994 [44]	Korea	1 case (M/45)	Lower back pain	Not reported	BSA	Bees	Not reported	Not reported	Not reported	Generalized erythematous plaques	Moderate	SP	-	Contact urticaria	Probable
Yoon 2012 [45]	Korea	2 cases a) M/33	a) Lower back pain	a) KMD	a) BVA	Bees	a) Tested (negative)	a) BV injection 2,000:1, 0.4 mL	a) Not reported	a) Facial edema, generalized pruritus, erythema, respiratory depression, and fever	a) Severe	a) SR	a) Grade II	a) Hypersensitivity	a) Probable
		b) F/75	b) Facial palsy	b) KMD	b) BVA	Bees	b) Tested (negative)	b) BV injection 2,000:1, 0.4 mL	b) Acupuncture, pharmacopuncture, herbal medicine, physical therapy	b) Localized edema	b) Moderate	b) SP	b) -	b) Hypersensitivity	b) Probable
Youn 2005 [46]	Korea	2 cases a) F/66	a) Knee pain	a) KMD	a) BVA	Bees	a) Not reported	a) BV injection 2,000:1, 0.2 mL	a) Acupuncture, pharmacopuncture	a) Chest discomfort, nausea, dizziness, drowsiness, and chills	a) Moderate	a) SR	a) Grade II	a) Anaphylaxis	a) Probable
		b) M/39	b) Posterior neck and shoulder pain	b) KMD	b) BVA	Bees	b) Not reported	b) BV injection 2,000:1, 0.3 mL	b) Acupuncture, cupping	b) Chest discomfort, generalized erythema, pruritus, dyspnea, and nausea	b) Moderate	b) SR	b) Grade II	b) Anaphylaxis	b) Probable
Yu 1998 [47]	Korea	2 cases a) F/43	a) Pruritic skin eruption	a) Not reported	a) BSA	Bees	a) Not reported	a) Not reported	a) Not reported	a) Multiple erythematous plaques and nodules, tenderness	a) Moderate	a) SP	a) -	a) Foreign body granulomas	a) Possible
		b) M/50	b) Subcutaneous nodule	b) Not reported	b) BSA	Bees	b) Not reported	b) Not reported	b) Not reported	b) Ill-defined subcutaneous nodules	b) Moderate	b) SP	b) -	b) Foreign body granulomas	b) Possible
Zhang 1994 [48]	China	2 cases a) M/50	a) Knee joint soft tissue damage	a) Not reported	a) BSA	Bees	a) Not tested	a) Not reported	a) Not reported	a) Localized edema; two ecphymas	a) Moderate	a) SP	a) -	a) Live bee acupuncture dermatitis	a) Probable
		b) M/29	b) Lumbodorsal fibromyalgia	b) Not reported	b) BSA	Bees	b) Not tested	b) Not reported	b) Not reported	b) Generalized pruritus limb paralysis, dyspnea, nausea, vomiting, systemic papules, large amounts of sweat, paralysis, and tremors	b) Severe	b) SR	b) Grade III	b) Anaphylaxis	b) Probable
Zhong 2005 [49]	China	1 case (F/51)	Osteoarthritis pain	MD	BSA	Bees	Not reported	30–40 bee stings	Not reported	Anorexia, listlessness, jaundice	Severe	Others	-	Acute icteric hepatitis	Possible
**Venom immunotherapy (VIT)**															
Anfosso-Capra 1990 [50]	France	1 case (F/49)	Not reported	Not reported (performed at the hospital)	Rush VIT	Wasps	Tested (positive)	YJV 60 μg	Not reported	Urticaria and cough	Mild	SR	Grade I	Hypersensitivity	Probable
Bousquet 1988 [51]	France	4 cases a) M/42	a) Treatment of systemic allergic reactions	a) MD	a) VIT	Bees	a) Tested (positive)	a) HBV 50 μg	a) Not reported	a) Angioedema involving the larynx and tracheobronchial tree; hypotension	a) Severe	a) SR	a) Grade III	a) Anaphylaxis	a) Probable
		b) M/16	b) Treatment of systemic allergic reactions	b) MD	b) VIT	Bees	b) Tested (positive)	b) HBV 100 μg	b) Not reported	b) Urticaria, tracheobronchial angioedema, and slight hypotension	b) Moderate	b) SR	b) Grade II	b) Anaphylaxis	b) Probable
		c) M/26	c) Treatment of systemic allergic reactions	c) MD	c) VIT	Bees	c) Tested (positive)	c) HBV 100 μg	c) Not reported	c) Increased pulse rate and decreased blood pressure	c) Severe	c) SR	c) Grade III	c) Anaphylaxis	c) Probable
		d) M/19	d) Treatment of systemic allergic reactions	d) MD	d) VIT	Bees	d) Tested (positive)	d) HBV 100 μg	d) Not reported	d) Mild hypotension, tachycardia, severe headaches, and erythematous rash	d) Severe	d) SR	d) Grade III	d) Anaphylaxis	d) Probable
De Bandt 1997 [52]	France	1 case (M/69)	Desensitization of BV	MD	VIT	Wasps	Tested (positive)	Not reported	Not reported	Motor loss in the left upper limb, weakness of both lower limbs, high grade fever, generalized rash, an indurated erythematous skin lesion over the left forearm, and arthritis of both wrists	Severe	SR	Grade III	Serum sickness reaction	Probable
Eming 2004 [53]	Germany	1 case (F/51)	Desensitization of BV	Not reported	Rush VIT	Bees	Tested (positive)	Not reported	Not reported	Multiple erythematous and subcutaneous nodules	Moderate	SP	-	Panniculitis	Possible
Karakurt 2010 [54]	Turkey	1 case (f/45)	Desensitization of BV	MD	VIT	Bees	Tested (positive)	Not reported	Not reported	Painful cyclic uterine contractions	Moderate	Others	-	Hypocalcemia or electrolyte imbalance	Probable
Lyanga 1982 [55]	Canada	1 case (F/24)	Desensitization of BV	MD	VIT	Wasps	Tested (positive)	Vespid venom 0.433 μg–100 μg	Not reported	Transient bradycardia	Moderate	Others	-	Idiosyncratic or direct toxic effect	Probable
Nemat 2011 [56]	Germany	1 case (F/16)	Desensitization of BV	MD	VIT	Bees	Tested (positive)	Not reported	Not reported	Severe pain affecting the left shoulder, chest wall, and left arm; muscular weakness in left the arm and hand; shortness of breath	Severe	Others	-	Neuralgic amyotrophy	Possible
Pijak 2011 [57]	Slovak Republic	1 case (M/47)	Because of significant professional risk	Not reported	VIT	Wasps	Tested (positive)	Not reported	Not reported	Elevations of aminotransferases and development of nephrotic syndrome	Severe	Others	-	Hepatitis B reactivation complicated with nephrotic syndrome	Probable
Reisman 1988 [58]	USA	7 cases a) F/39	a) Desensitization of BV	a) MD	a) VIT	Mix	a) Tested (positive)	a) HBV 1.0 μg, YJV 0.1 μg, *Polistes* venom 0.1 μg	a) Not reported	a) Nausea, emesis, headache, fever, malaise	a) Moderate	a) SR	a) Grade II	a) Late onset reaction	a) Possible
		b) M/40	b) Desensitization of BV	b) MD	b) VIT	Mix	b) Tested (positive)	b) HBV 5.0 μg, YJV 2.0 μg	b) Not reported	b) Fatigue, malaise, local swelling	b) Moderate	b) SR	b) Grade I	b) Late onset reaction	b) Possible
		c) M/70	c) Desensitization of BV	c) MD	c) VIT	Mix	c) Tested (positive)	c) HBV 50.0 μg, YJV 5.0 μg	c) Not reported	c) Generalized aches, joint pain	c) Moderate	c) Others	c) -	c) Late onset reaction	c) Possible
		d) M/37	d) Desensitization of BV	d) MD	d) VIT	Mix	d) Tested (positive)	d) HBV 0.3 μg, YJV 0.3 μg	d) Not reported	d) Muscle aches, joints sore, difficulty in walking	d) Moderate	d) Others	d) -	d) Late onset reaction	d) Possible
		e) F/41	e) Desensitization of BV	e) MD	e) VIT	Wasps	e) Not reported	e) YJV 50.0 μg	e) Not reported	e) Chills, fever, aches	e) Moderate	e) SR	e) Grade I	e) Late onset reaction	e) Possible
		f) M/50	f) Desensitization of BV	f) MD	f) VIT	Wasps	f) Tested (positive)	f) YJV 50.0 μg	f) Not reported	f) Asthma, chest tightness	f) Moderate	f) Others	f) -	f) Late onset reaction	f) Possible
		g) M/27	g) Desensitization of BV	g) MD	g) VIT	Mix	g) Tested (positive)	g) HBV 0.1 μg, YJV 0.1 μg	g) Not reported	g) Generalized ache, fatigue	g) Moderate	g) SR	g) Grade I	g) Late onset reaction	g) Possible
Yalcin 2012 [59 ^]^	Turkey	1 case (M/61)	Desensitization of BV	MD	VIT	Bees	Tested (positive)	Not reported	Not reported	Severe itching, erythematous papules, and plaques	Moderate	SP	-	Jessner lymphocytic infiltrate	Possible

AE: adverse event; BVT: bee venom therapy; BSA: bee sting acupuncture; BVA: bee venom acupuncture; SBV: sweet bee venom; HBV: honeybee venom; KMD: Korean medical doctor; MD: Medical doctor; VIT: venom immunotherapy; YJV: yellow jacket venom.

^a^ Venom type: bees (family Apidae); wasps (family Vespidae); mix (bees and wasps).

^b^ AE severity was assessed using Spilker’s criteria: mild, moderate, and severe.

^c^ AE type was classified into 1 of 3 categories: systemic reaction (SR), skin problem (SP), and other.

^d^ Mueller classification: if a systemic reaction occurred as an AE, it was classified into 1 of 5 categories: large local reaction, grade I, grade II, grade III, and grade IV.

^e^ Causality was determined through the WHO-UMC causality scale: certain, probable, possible, unlikely, conditional, and inaccessible.

### Audits and cohort studies

AEs were also reported in 79 mainly retrospective audit studies that aimed to assess the safety of BVT (Table 2) [60–138]. These studies were chiefly observational and included case-controlled and cohort studies. VIT (63 studies) was the most commonly used BVT method, followed by BSA (9 studies) and BVA (7 studies). The treatment protocol for VITs included conventional VIT, cluster VIT, rush VIT, ultra-rush VIT, specific immunotherapy, and rush-specific immunotherapy. Eleven studies were conducted in Spain, 10 studies were conducted in China, 8 studies were conducted in Italy and the United States, 6 studies were conducted in Germany, 5 studies were conducted in France, Korea, and Switzerland, and 21 studies were conducted in 18 other countries. The prevalence of AEs ranged from 0.00% [60] [117] [118] [134] up to 90.63% [109]. In the 46 VIT studies, the median incidence (number of patients with AEs/number of patients in all cases, %) of AEs was 28.87% (interquartile range [IQR], 14.57–39.74%), and the AE types included SR (50.37%), LR (35.80%), LLR (9.99%), and other (3.85%; blood pressure elevation, moderate hypotension, rhinitis, asthenia or headache, visual disorders and vertigo, transient dyspnea, proteinuria with microscopic hematuria, generalized pruritus without skin lesions or other signs, and not reported).

**Table 2:** Audits and cohort studies on the adverse events of bee venom therapy .

Study (first author, year)	Country	Prospective or retrospective study^a^	Stimulation features of bee venom therapy	Venom type^b^	Incidence of AEs^c^	Types of AEs (numbers or cases)
**Bee sting acupuncture (BSA) and bee venom acupuncture (BVA)**						
Castro 2005 [60]	USA	Prospective study	Bee venom acupuncture	Bees	0/9 (0.00%)	LR (minor)
Choi 2010 [61]	Korea	Retrospective study	SBV	Bees	48/374 (12.83%)	LR (48)
Gao 2011 [62]	China	Retrospective study	Bee sting acupuncture	Bees	395/250^d^	-
Hwang 2000 [63]	Korea	Retrospective study	Bee venom acupuncture	Bees	11/32,000 (0.03%)^g^	SR (11)
Jung 2013 [64]	Korea	Retrospective study	SBV	Bees	a) 37/130 (28.46%) b) 41/130 (31.54%)	a) LR (37) b) LR (41)
Kwon 2000 [65]	Korea	Retrospective study	Bee venom acupuncture	Bees	361/2765 (13.00%)^e^	SR (361)
Li 1995 [66]	China	Retrospective study	Bee sting acupuncture	Bees	186/160^d^	-
Liu 1993 [67]	China	Retrospective study	Bee sting acupuncture	Bees	96/32^d^	-
Ma 2008 [68]	China	Retrospective study	Bee venom acupuncture	Bees	7/40^d^	-
Tang 2003 [69]	China	Retrospective study	Bee sting acupuncture	Bees	20/468 (4.27%)	SR (20)
Wen 2003 [70]	China	Retrospective study	Bee sting acupuncture	Bees	12/40^d^	-
Xiao 2013 [71]	China	Retrospective study	Bee sting acupuncture	Bees	4902/4960 (98.83%)^e^	LR (4902)
Yoon 2012 [72]	Korea	Prospective study	SBV	Bees	2/11 (18.18%)	LR (2)
Yu 2006 [73]	China	Retrospective study	Bee sting acupuncture	Bees	30/250 (12.00%)	SR (30)
Zhang 2010 [74]	China	Retrospective study	Bee sting acupuncture	Bees	141/120^d^	-
Zhou 2009 [75]	China	Retrospective study	Bee sting acupuncture	Bees	3/40 (7.50%)	SR (3)
Venom immunotherapy (VIT)						
Aguilar 1999 [76]	Spain	Retrospective study	CVIT	Mix	12/70 (17.14%)	SR (4), LR (5), Others (3)
Alessandrini 2006 [77]	Italy	Prospective study	VIT	Wasps	40/107 (37.38%)	SR (7), LR (33)
Anguita Carazo 2011 [78]	Spain	Retrospective study	VIT	Hymenoptera	35/2,935 (1.19%)^e^	SR (9), LR (26)
				Bees	12/1291 (0.93%)	SR (9), LR (3)
				Wasps	23/1644 (1.40%)	SR (0), LR (23)
Bemanian 2007 [79]	Iran	Prospective study	CVIT	Mix	8/120 (6.66%)^e^	SR (8)
Bernstein 1989 [80]	USA	Retrospective study	Rapid VIT	Single or mix	19/33 (57.58%)	SR (4), LR (18)
Bernstein 1994 [81]	USA	Retrospective study	VIT	Single or mix	4/77 (5.19%)	SR (4)
Birnbaum 1993 [82]	France	Retrospective study	RVIT	Hymenoptera	34/284 (11.97%)	SR (34)
				Bees	24/91 (26.37%)	SR (24)
				Wasps	10/193 (5.18%)	SR (10)
Birnbaum 2003 [83]	France	Retrospective study	Ultra-RVIT	Single or mix	36/325 (11.08%)	SR (36)
Bonadonna 2008 [84]	Italy	Retrospective study	SIT	Single	2/16 (12.50%)	SR (1), Others (1)
Bonadonna 2013 [85]	Italy and Spain	Prospective study	VIT	Single or mix	10/84 (11.90%)	SR (4), LLR (6)
Brehler 2000 [86]	Germany	Retrospective study	VIT	Single	224/1,055 (21.23%)^e^	SR (160), LR (124)
Bucher 2003 [87]	Switzerland	Retrospective study	Ultra-RVIT	Hymenoptera	127/179 (70.95%)	SR (24), LR (103)
				Bees	63/85 (74.12%)	SR (18), LR (45)
				Wasps	64/94 (68.09%)	SR (6), LR (58)
Cadario 2004 [88]	Italy	Prospective study	VIT	Single	15/45 (33.33%)	SR (4), LR (11)
Calaforra 2009 [89]	Spain	Retrospective study	CVIT	Single	22/863 (2.55%)^e^	SR (15), LR (7)
Carballada 2003 [90]	Spain	Retrospective study	VIT	Single	52/241 (21.58%)	SR (22), LR (31)
Carballada Gonzalez 2009 [91]	Spain	Retrospective study	VIT	Hymenoptera	5/21 (23.81%)	SR (2), LR (3)
				Bees	5/17 (29.41%)	SR (2), LR (3)
				Wasps	-0/4 (0.00%)	
Catalá 2009 [92]	Spain	Retrospective study	CVIT	Single	7/180 (3.89%)^e^	SR (2), LLR (3), Others (2)
Caubet 2008 [93]	Switzerland	Retrospective study	Subcutaneous IT	Hymenoptera	173/1,278 (13.54%)^e^	SR (53), LLR (120)
Cavallucci 2010 [94]	Italy	Retrospective study	VIT	Single	a) IP 32/72 (44.44%)	a) SR (9), LR (23)
					b) EP 22/72 (30.56%)	b) SR (4), LR (18)
					c) MP 17/72 (23.61%)	c) SR (0), LR (17)
De Jong 1999 [95]	Netherlands	Retrospective study	VIT	Bees	14/194 (7.22%)^e^	SR (2), LR (12)
Dursun 2006 [96]	Turkey	Retrospective study	VIT	Mix	2/20 (10.00%)	SR (2)
Eben 2010 [97]	Germany	Retrospective study	VIT	Single	54/159 (33.96%)	SR (36), Others (18)
Gastaminza 2003 [98]	Spain	Retrospective study	VIT	Mix	<250/4973 (<5.03%)^e^ ^,^ ^f^	SR (<79)
Goldberg 2011 [99]	Israel	Retrospective study	RVIT	Single or mix	53/179 (29.61%)	SR (53)
Golden 1980 [100]	USA	Retrospective study	Slow VIT, RVIT, or Step VIT	Single or mix	42/64 (65.63%)	SR (10), LLR (32)
Golub 1984 [101]	USA	Retrospective study	VIT	Single or mix	10/41 (24.39%)	SR (1), LR (9)
Gonzalez de Olando 2008 [102]	Spain	Retrospective study	VIT	Single or mix	6/21 (28.57%)	SR (6)
Gorska 2008 [103]	Poland	Retrospective study	RVIT	Hymenoptera	18/118 (15.25%)	SR (18)
				Bees	8/28 (28.57%)	SR(8)
				Wasps	10/90 (11.11%)	SR(10)
Hirata 2003 [104]	Japan	Retrospective study	RVIT	Single or mix	3/95 (3.16%)	SR (3)
Kerddonfak 2009 [105]	Thailand	Retrospective study	RVIT	Single or mix	<4/6 (<66.67%)^f^	SR (<3), LLR (<1)
Kalogeromitros 2009 [106]	USA	Prospective study	RVIT	Single or mix	9/49 (18.37%)	SR (9)
Köhli-Wiesner 2012 [107]	Switzerland	Retrospective study	Ultra-RVIT	Single or mix	16/94 (17.02%)^e^	SR (13), Others (3)
Kopaè 2009 [108]	Slovenia	Retrospective study	Ultra-RVIT	Single	14/77 (18.18%)	SR (10), LLR (4)
Lata 2005 [109]	Poland	Retrospective study	SIT	Mix	29/32 (90.63%)	SR (6), LR(23)
Laurent 1997 [110]	France	Retrospective study	RVIT	Single or mix	39/97 (40.21%)	LLR (9), Others (30)
Lee 2006 [111]	Germany	Prospective study	Ultra-RVIT	Wasps	28/110 (25.45%)	SR (5), LLR (23)
Marquès 2010 [112]	Spain	Retrospective study	VIT	Single or mix	184/536 (34.33%)	SR (35), LR (149)
Mellerup 2000 [113]	Denmark	Retrospective study	VIT	Mix	14/117 (11.97%)	SR (14)
Mingomataj 2002 [114]	Albania	Retrospective study	RSIT	Single	16/37 (43.24%)	SR (16)
Mosbech 2000 [115]	10 European countries	Prospective study	VIT	Single or mix	20.00%	-
Müller 1992 [116]	Switzerland	Retrospective study	RVIT or VIT	Hymenoptera	74/205 (36.10%)	SR (74)
				Bees	60/148 (40.54%)	SR (60)
				Wasps	14/57 (24.56%)	-R (14)
Nagai 2004 [117]	Japan	Retrospective study	RVIT	Mix	0/2 (0.00%)	-
Nataf 1984 [118]	France	Retrospective study	RVIT	Mix	0/54 (0.00%)^g^	-
Pasaoglu 2006 [119]	Turkey	Retrospective study	RVIT	Hymenoptera	15/469 (3.20%)^e^	SR (4), LR(11)
				Bees	12/240 (5.00%)	SR (4), LR(8)
				Wasps	3/229 (1.31%)	LR(3)
Poli 2001 [120]	Italy	Retrospective study	VIT	Wasps	2/36 (5.56%)	LR (2)
Quercia 2001 [121]	Italy	Retrospective study	RVIT or CVIT	Bees	17/55 (30.91%)	SR (8) LLR (9)
Quercia 2006 [122]	Italy	Prospective study	VIT or CVIT	Bees	a) IP 20/68 (29.41%)	a) SR (9) LR (11)
					b) MP 5/68 (7.35%)	b) SR (5)
Ramirez 1981 [123]	USA	Retrospective study	VIT	Hymenoptera	36/859 (4.19%)^e^	LLR(36)
Rocklin 1982 [124]	USA	Retrospective study	VIT	Single	1/1032 (0.01%)^e^	SR(1)
Roll 2006 [125]	Switzerland	Retrospective study	Ultra-RVIT	Single or mix	14/80 (17.50%)	SR (10), LLR (4)
Roumana 2009 [126]	Greece	Retrospective study	RVIT or Ultra-RVIT	Single or mix	219/8,030 (2.73%)^e^	SR (219)
Ruëff 1997 [127]	Germany	Retrospective study	RVIT	Hymenoptera	57/144 (39.58%)	SR (57)
				Bees	11/28 (39.29%)	SR (11)
				Wasps	46/116 (39.66%)	SR (46)
Ruëff 2004 [128]	Germany	Prospective study	SIT	Bees	a) IP 39/65 (60.00%)	a) SR (16), LLR (23)
					b) MP33/46 (71.74%)	b) SR (8), LLR (25)
Sánchez-Machín 2010 [129]	Spain	Retrospective study	CVIT	Bees	25/54 (46.30%)	SR(2), LR (23)
Sánchez-Morillas 2005 [130]	Spain	Retrospective study	RVIT	Single or mix	14/48 (29.17%)	SR (2), LR (12)
Schiavino 2004 [131]	Italy	Retrospective study	Ultra-RVIT	Hymenoptera	20/57 (35.09%)	SR (4) LR (16)
				Bees	5/9 (55.56%)	SR (1) LR (4)
				Wasps	15/48(31.25%)	SR (3) LR (12)
Sporcic 2009 [132]	Serbia and Montenegro	Retrospective study	VIT	Single or mix	6/14 (42.86%)	SR (2), LR (4)
Sturm, 2002 [133]	Austria	Retrospective study	RVIT	Single	7/101 (6.93%)	SR (7)
Tarhini 1992 [134]	France	Prospective study	CVIT	Single or mix	0/100 (0.00%)	-
Thurnheer 1983 [135]	Sweden	Retrospective study	RVIT or VIT	Single or mix	24/42 (57.14%)	SR (16) LLR (8)
Wenzel 2003 [136]	Germany	Retrospective study	RVIT	Single or mix	32/178 (17.98%)	SR (32)
Westall 2001 [137]	Australia	Retrospective study	RVIT	Hymenoptera	26/68 (38.24%)	SR (26)
				Bees	25/60 (41.67%)	SR (25)
				Wasps	1/8 (12.50%)	SR (1)
Youlten 1995 [138]	UK	Retrospective study	VIT	Hymenoptera	24/109 (22.02%)	SR (24)
				Bees	12/83 (14.46%)	SR (12)
				Wasps	12/26 (46.15%)	SR (12)

AE: adverse event; SR: systemic reaction; LR: local reaction; LLR: large local reaction; VIT: venom immunotherapy; RVIT: rush VIT; SIT: specific immunotherapy; RSIT: rush-specific immunotherapy; CVIT; cluster VIT; IP: induction phase; EP: extension phase; MP: maintenance phase.

^a^ If it was not reported in prospective articles, it was considered a retrospective study.

^b^ Venom type: bees (family Apidae); wasps (family Vespidae); single (some bee venom or some wasp venom); mix (bee and wasp venom).

^c^ Incidence: number of patients with AEs/number of patients of total cases, %

^d^ Incidence: number of cases with AEs/number of patients of total cases.

^e^ Incidence: number of injections (dose) that resulted in AEs/total number of injections (dose), % (if the number of patients with AEs was not mentioned or precisely presented).

^f^ Incidence of AEs caused by BVTs combined with the incidence of AEs from other allergens.

^g^ This study was the only report of anaphylaxis related to BVT.

### RCTs and randomized crossover trials

Eighteen RCTs and 2 randomized crossover trials were included in this review (Table 3) [139–158]. One-hundred and forty-eight AEs related to BVT were reported in 397 participants. Seventeen patients ended their study participation owing to BVT-related AEs. For the BSA and BVA studies, all of the participants who were negative for skin allergy tests were included in the studies. With regard to the quality of the reporting of AEs, more than half of the items in the CONSORT AE reporting guidelines were not reported (52.14%). Most RCTs did not report the AEs in the title, abstract, or introduction, or report definitions of AEs and mention the methods for analyzing and presenting AEs. In 9 studies, the collecting and monitoring method for AEs involved retrospectively checking with the physician and/or participant, and the monitoring methods of 7 studies were not reported appropriately. Most studies reported the number of patients who stopped participating, as well as the specific denominator for the analysis of BVT-related AEs.

**Table 3:** Randomized controlled trials and randomized crossover trials reporting adverse events of bee venom therapy.

Study (first author, year)	Disease type	Intervention	Control	Skin test	Incidence and type of AEs^a^		Quality of AE reporting (CONSORT items for reporting AEs)^b^						
					Bee venom therapy	Control	1	2	3	4	5	6	7
**Bee sting acupuncture (BSA) and bee venom acupuncture (BVA)**													
Cho 2012 [139]	Idiopathic Parkinson’s disease (RCT)	BVA	Acupuncture; no treatment	Tested (negative)	0/18	0/17; 0/14	Not reported	Not reported	Not reported	Moderate (retrospective checking by participant)	Not reported	Moderate (1 drop-out because of pruritus)	Moderate
Cho 2013 [140]	Central post-stroke pain (RCT)	BVA	Normal saline injection	Tested (negative)	0/10	0/10	Not reported	Not reported	Not reported	Bad	Not reported	Moderate (1 drop-out because of pruritus)	Moderate
Deng 2011 [141]	Rheumatoid arthritis (RCT)	BSA	Methotrexate; Prednisone and methotrexate	Tested (negative)	5/20 (localized swelling and pruritus, fever (3), nausea (2))	4/20 (nausea (3), leukopenia (1)); 9/20 (nausea, flatulence (6), mental excitation, insomnia (3))	Moderate	Bad	Not reported	Moderate (retrospective checking by physician)	Good	Not reported	Good
Gwak 2009 [142]	Central post-stroke (RCT)	BVA	Normal saline injection	Tested (negative)	Not reported	Not reported	Not reported	Not reported	Not reported	Moderate (retrospective checking by participant)	Not reported	Moderate (1 drop-out because of pruritus)	Not reported
Kim 2005 [143]	Sprain of C-spine (RCT)	BVA and acupuncture	Normal saline injection and acupuncture	Tested (negative)	Not reported	Not reported	Not reported	Not reported	Bad	Bad	Not reported	Moderate (1 drop-out because of hypersensitivity)	Not reported
Ko 2007 [144]	Shoulder pain after stroke (RCT)	BVA	Normal saline injection	Tested (negative)	13/24 (pruritus (8), burning, sensation (3), pain (2))	6/22 (pruritus (2), burning sensation (1), pain (3))	Not reported	Not reported	Bad	Moderate (retrospective checking by physician)	Not reported	Not reported	Good
Koh 2013 [145]	Adhesive capsulitis (RCT)	BVA and physiotherapy	Normal saline injection and physiotherapy	Tested (negative)	31/45 (slight pruritus, local swelling, and/or redness (30), mild, generalized swelling and aching (1))	3/23 (slight redness and pruritus)	Not reported	Not reported	Bad	Moderate (retrospective checking by physician)	Not reported	Moderate (1 drop-out because of allergic response)	Good
Ku 2010 [146]	Carpal tunnel syndrome (RCT)	BVA	Scolopendrid pharmacopuncture	Tested (negative)	0/11	Not reported	Moderate	Not reported	Bad	Bad	Not reported	Moderate (1 drop-out because of allergic response)	Bad
Lee 2003 [147]	Rheumatoid arthritis (RCT)	BVA	Normal saline injection	Tested (negative)	Not reported	Not reported	Not reported	Not reported	Not reported	Bad	Not reported	Moderate (2 drop-out because of pruritus)	Not reported
Noh 2010 [148]	Upper limb spasticity after stroke (randomized crossover trial)	BVA	Normal saline injection	Tested (negative)	Not reported	Not reported	Not reported	Not reported	Not reported	Not reported	Not reported	Moderate (2 drop-out because of pruritus)	Not reported
Rong 2002 [149]	Rheumatoid arthritis (RCT)	BSA	Methotrexate, auranofin, and NSAIDs	Tested (negative)	3/20 (fever, localized erythema (3))	9/20 (stomach discomfort and pain, nausea, loss of appetite, diarrhea, mouth dry, rash (9))	Not reported	Bad	Bad	Moderate (retrospective checking by physician)	Moderate	Moderate (no drop-out)	Good
Shin 2012 [150]	Chronic low back pain (RCT)	BVA	Normal saline injection	Tested (negative)	17/30 (pruritus (15), erythema (5), edema (4), skin rash (2))	3/30 (skin rash (1), headache (1), hand and foot tingling (1))	Moderate	Not reported	Good	Good (retrospective checking by physician, research coordinator, and participant)	Good	Moderate (1 drop-out because of pruritus)	Good
Song 2005 [151]	Acute ankle sprain (RCT)	BVA	Normal saline injection	Not reported	Not reported	Not reported	Not reported	Not reported	Not reported	Bad	Not reported	Moderate (1 drop-out because of pruritus)	Not reported
Wen 2011 [152]	Ankylosing spondylitis (RCT)	BSA	Sulfasalazine	Tested (negative)	4/40 (pruritus, skin eruption (3), slight fever (1))	10/40 (epigastric discomfort slight pain, nausea (9), hepatic function abnormal (3), leukopenia (1), drug hypersensitivity syndrome (1))	Good	Bad	Bad	Not reported	Not reported	Not reported	Good
Wen 2012 [153]	Ankylosing spondylitis (RCT)	BSA	Sulfasalazine	Tested (negative)	4/30 (pruritus, skin eruption (3), slight fever (1))	12/30 (epigastric discomfort slight pain, nausea (7), hepatic function abnormal (2), leukopenia (2), drug hypersensitivity syndrome (1))	Good	Not reported	Not reported	Not reported	Not reported	Not reported	Good
Wesselius 2005 [154]	Multiple sclerosis (randomized crossover trial)	BVT	No treatment	Tested (negative)	11/26 (extreme localized swelling (2), pruritus (4), flulike symptoms (5), no serious AEs)	0/26	Moderate	Not reported	Not reported	Moderate (retrospective checking by physician and participant)	Not reported	Not reported	Good
Won 1999 [155]	Knee or spinal osteoarthritis (RCT)	BVA	Nabumetone	Tested (negative)	60/76 (pruritus (60), chill and pain (49), local pain (36), edema (25), muscle pain (16), headache (14), fever (13), nausea (4), sweating (3), fatigue (3), vertigo (3), vomiting (1), abdominal pain (1))	Not reported	Good	Good	Good	Moderate	Good	Moderate (2 drop-out because of blisters (1) and urticaria (1))	Good
Yoo 2008 [156]	Cancer-related pain (RCT)	SBV	Normal saline injection	Tested (negative)	Not reported	Not reported	Not reported	Not reported	Not reported	Bad	Not reported	Moderate (1 drop-out because of pain aggravation)	Not reported
Venom immunotherapy (VIT)													
Oude Elberink 2002 [157]	Desensitization of BV (RCT)	VIT (YJV)	EpiPen	Tested (positive)	Not reported	Not reported	Not reported	Not reported	Good	Moderate (retrospective checking by physician)	Not reported	Bad (2 drop-out because of AEs)	Moderate
Oude Elberink 2006 [158]	Desensitization of BV (RCT)	VIT (YJV)	EpiPen	Tested (positive)	0/47 (no systemic AEs reported)	Not reported	Not reported	Not reported	Not reported	Bad	Not reported	Not reported	Moderate

AE: adverse event; BVT: bee venom therapy; BSA: bee sting acupuncture; BVA: bee venom acupuncture; SBV: sweet bee venom; VIT: venom immunotherapy; YJV: yellow jacket venom. Quality of reporting: good, clear, and well described; moderate, described but not in detail; bad, inappropriately described; not reported, not described at all.

^a^ Incidence: number of patient with AEs/number of patients of total cases, %.

^b^ CONSORT items for reporting AEs: 1, statement of AEs in title or abstract; 2, statement of BVT related AEs in the introduction; 3, predefined definition of AEs related to the BVT; 4, collection or monitoring method for AEs; 5, statement of the method for analyzing and presenting AEs; 6, statement of any patients who dropped out due to AEs; 7, description of the specific denominator for the analysis of AEs.

**Fig 2:**
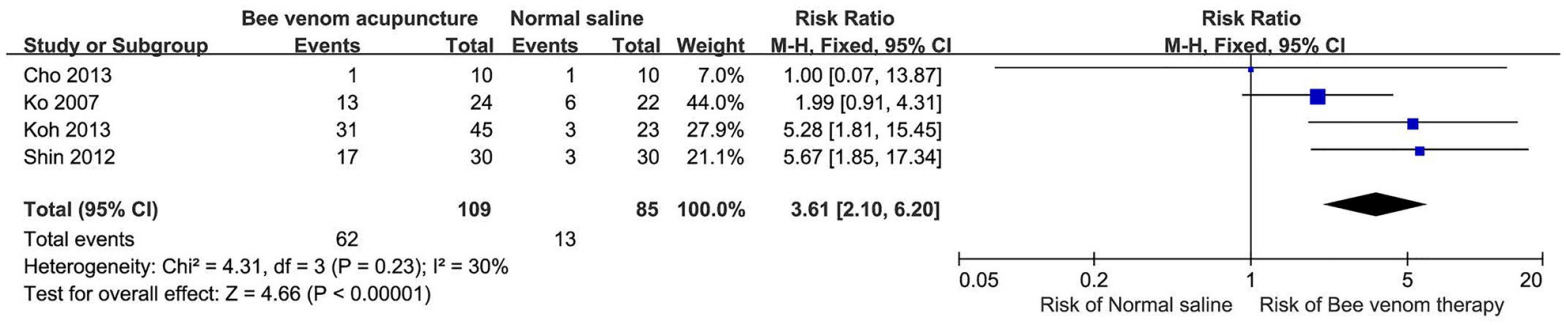
Relative risk of adverse events in randomized controlled trials with bee venom therapy and saline.

The meta-analysis of AE occurrence in the 4 RCTs assessing patients experiencing AEs showed that BVA increased the risk of AEs by 261% compared to the risk associated with normal saline control treatment (relative risk, 3.61; 95% CI [2.10, 6.20], Fig 2).

## Discussion

The aim of our systematic review was to summarize the evidence pertaining to BVT-related AEs by analyzing AE types and their prevalence in patients. We reviewed 145 studies, including 20 RCTs and randomized crossover studies, 79 audits and cohort studies, 33 single-case studies, and 13 case series. According to our findings, BVT can lead to AEs such as SRs, LLRs, LRs, SPs, and nonspecific reactions, some of which are serious.

In case studies and case series, we found that SRs comprised 51.72% of the AEs produced by bee venom. Moreover, the identified severe AEs included 14 cases of grade III SR and 1 case of grade IV SR (50.00% of the total SRs). We also found that there have been SAEs associated with BVT that urgently required subcutaneous adrenaline or steroid and oxygen therapy, with death occurring in 1 case [22] [51] [58]. Aside from SRs, AEs associated with BSA and BVA mainly include SPs such as granulomas and plaques, which may be attributable to persistent local inflammation caused by venomic components or from the remaining stinger at the site of cutaneous injection [29]. In contrast, SRs resulting from BVT are mainly derived from anaphylaxis, hypersensitivity, and late-onset reactions [3] [58].

In 46 audits and cohort studies of VIT, the median incidence of AEs was 28.87%, and SRs occurred in 681/4844 (14.06%) participants. These results suggest a more frequent AE incidence in comparison with that in previous systematic reviews of VIT, which reported SR incidences of 11.5 to 11.8% [159] [160]. Interestingly, some studies found a complete lack of AEs related to BVT and a corresponding lack of SRs, and some studies have shown minor AEs, but no serious SRs [60] [117,118] [134].

Skin tests allow practitioners to distinguish whether BVT is an appropriate intervention for particular patients. In most RCTs and randomized crossover trials with BSA and BVA, participants were included if they showed negative responses in skin tests, whereas participants were included in VIT case studies and case series if they showed positive responses in skin tests. This difference in the participants included in each type of study does not seem to be directly related to the AEs associated with BVT; negative venom skin test results are not always a guarantee of VIT safety [94]. However, serious AEs can occur as a result of BSA and BVA in patients with positive skins tests. There is a report of a young, healthy adult who was sensitized to bee venom through BSA, and who was later stung by a bee and developed severe, life-threatening anaphylaxis [161].

Venom concentration and the frequency of venom administration can influence the severity and rate of incidence of AEs resulting from BSA and BVA. Unfortunately, we could not analyze the effect of venom concentration and administration frequency on the severity and rate of incidence of AEs because only limited numbers of RCTs were included in this review.

With regard to the quality of reporting of AEs in RCTs, CONSORT items were generally not reported properly. Future RCTs with BVT must adopt the CONSORT AE reporting guidelines to ensure transparency and accuracy. When designing protocols, methods of AE assessment based on the CONSORT AE reporting guidelines should be suggested in detail.

AEs related to BVA or VIT have been reported in various studies, including surveys [8] [162] [163] and reviews [159] [160] [164] [165]. However, in this paper, we extensively reviewed all types of BVT (BSA, BVA, SBV, apitoxin injection, and VIT). We focused on the incidence of AEs in audit and cohort studies related to BVT, and sought to provide an overview of the many types of AEs that were reported in case studies and case series. We performed this investigation through a comprehensive search of the literature.

This review has some limitations. First, the heterogeneity of intervention in the reviewed articles was high; thus, the exact AE incidence and risk associated with the treatment methods could not be calculated. Second, although different venoms were used in different therapies (bee [family Apidae] venom was mainly used in BSA and BVA, whereas venom of both bees [family Apidae] and wasps [family Vespidae] was used in VIT), AEs from VIT were not classified in terms of the type of venom, treatment protocol (conventional VIT, cluster VIT, rush VIT, ultra-rush VIT, etc.), or phase (induction and maintenance).

While it is evident that BVA clearly increases the risk of AEs in comparison with normal saline, our review revealed that BSA and BVA are often implemented without a skin test, and also showed that patients have experienced SAEs that can be fatal after receiving BSA from unqualified personnel. Therefore, in order to enhance the safety of BVT, a skin test should be conducted before BVT is administered, and the venom should be administered only by qualified individuals [166].

Based on the results of this review, several suggestions can be made to support effective clinical practice and future clinical trials with BVT. In order to support responsible use of BVT, educational materials on the safety and efficacy of BVT should be made available for patients. Moreover, practitioners should be aware of the various AEs associated with BVT, establish clinical guidelines to minimize the development of AEs, and develop and implement strict criteria for monitoring AEs once they occur.

## Conclusion

AEs related to BVT are not uncommon. Therefore, BVT practitioners should pay careful attention to the incidence of AEs and patterns of AE occurrence in their patients. Additionally, education and qualification of BVT practitioners should be ensured based on appropriate training programs and clinical guidelines for monitoring of AEs related to BVA and BSA. Furthermore, when reporting AEs in RCTs evaluating BVT, researchers should describe AEs in detail according to the CONSORT recommendation for harm data to ensure transparency and accuracy.

## Supporting Information

S1 File Search strategies for the electronic databases. (DOCX)

S2 File PRISMA Checklist. (DOC)
