# Supplementary material for: Risk Associated with Bee Venom Therapy: A Systematic Review and Meta-Analysis
Source: PLoS One. 2015 May 21;10(5):e0126971. doi: 10.1371/journal.pone.0126971 (PMC4440710; doi:10.1371/journal.pone.0126971)
Supplement: S1 File — (DOCX) [file pone.0126971.s001.docx]

Supporting Information

Search strategies

**PubMed**

1 Bee Venoms [MeSH Terms]

2 bee sting

3 bee venom

4 sweet bee venom

5 bee sting acupuncture

6 bee sting therapy

7 honeybee acupuncture

8 bee venom therapy

9 bee venom acupuncture

10 bee acupuncture therapy

11 self-administered bee sting

12 honey bee venom acupuncture

13 bee venom injection

14 live bee acupuncture

15 Bong-chim

16 apitoxin

17 apitherapy [MeSH Terms]

18 Hymenoptera [MeSH Terms]

19 Venom Immunotherapy

20 hymenoptera venom

21 1 OR 2 OR 3 OR 4 OR 5 OR 6 OR 7 OR 8 OR 9 OR 10 OR 11 OR 12 OR 13 OR 14 OR 15 OR 16 OR 17 OR 18 OR 19 OR 20

22 Drug-Related Side Effects and Adverse Reactions [MeSH Terms]

23 adverse effects [MeSH Subheading]

24 Malpractice [MeSH Major Topic]

25 safe

26 safety

27 adverse event

28 adverse reaction

29 side effects

30 complications

31 risk

32 22 OR 23 OR 24 OR 25 OR 26 OR 27 OR 28 OR 29 OR 30 OR 31

33 21 AND 32

**Chinese Journals (CNKI, Wanfang, and Weipu)**

(蜂毒 OR 蜂毒疗法 OR 蜂毒穴位注射 OR 蜂针) AND (副作用 OR 反作用 OR 反效应 OR 治疗不当 OR 有害反应 OR 逆反应 OR 安全 OR 危害 OR 冒险 OR 过敏')

**Korean Journals (KoreaMED, KMBASE, KISS, NDSL, and OASIS)**

벌침 OR 봉침 OR 봉약침 OR 벌독 OR 봉독 OR 건조밀봉독 OR sweet bee venom

OR sweet BV
